# A guide to selecting high-performing antibodies for Secreted frizzled-related protein 1 (sFRP-1) for use in Western Blot and immunoprecipitation

**DOI:** 10.12688/f1000research.130991.2

**Published:** 2024-04-02

**Authors:** Riham Ayoubi, Kathleen Southern, Carl Laflamme, NeuroSGC/YCharOS collaborative group

**Affiliations:** 1 Department of Neurology and Neurosurgery, Structural Genomics Consortium, The Montreal Neurological Institute, McGill University, Montreal, Quebec, H3A 2B4, Canada

**Keywords:** Uniprot ID Q8N474, SFRP1, Secreted frizzled-related protein 1, antibody characterization, antibody validation, Western Blot, immunoprecipitation

## Abstract

Secreted frizzled-related protein 1 (sFRP-1) is a secreted protein, belonging to the secreted glycoprotein SFRP family. As a modulator of the Wnt/β-catenin signalling pathway, sFRP-1 has implications in human cancers and neurological diseases. If the community had access to well-characterized anti-sFRP-1 antibodies, the reproducibility of sFRP-1 research would be enhanced. In this study, we characterized 11 sFRP-1 commercial antibodies for Western Blot and immunoprecipitation, using a standardized experimental protocol based on comparing read-outs in knockout cell lines and isogenic parental controls. These studies are part of a larger, collaborative initiative seeking to address the antibody reproducibility issue by characterizing commercially available antibodies for human proteins and publishing the results openly as a resource for the scientific community. While use of antibodies and protocols vary between laboratories, we encourage readers to use this report as a guide to select the most appropriate antibodies for their specific needs.

## Introduction

SFRP-1 is a secreted protein belonging to the secreted frizzled-related protein (SFRP) family. ^1^ Having an amino acid terminal cysteine-rich domain homologous to the putative Wnt-binding domain of frizzled receptors, sFRP-1 functions as a modulator of the Wnt/β-catenin signalling pathway that is essential to multiple cellular processes. ^2^^,^^3^

Dysregulated Wnt/β-catenin activity levels play a role in various disease processes. ^4^ Epigenetic silencing of *SFRP1* elevates Wnt/β-catenin activity, which has been linked to cancer. ^2^^,^^4^ Conversely, high concentrations of sFRP-1 suppresses Wnt/β-catenin activity, which is predicted to have implications in neurological diseases, such as Alzheimer’s disease (AD). ^1^^,^^3^^–^^5^ Targeting sFRP-1 to restore the Wnt/β-catenin signalling pathway is of current interest in AD research to discover novel therapies. ^3^ Mechanistic studies would be greatly facilitated with the availability of high-quality antibodies.

This research is part of a broader collaborative initiative in which academics, funders and commercial antibody manufacturers are working together to address antibody reproducibility issues by characterizing commercial antibodies for human proteins using standardized protocols, and openly sharing the data. ^6^^–^^8^ Here, we compared the performance of a range of commercially available antibodies for sFRP-1 use in Western Blot and immunoprecipitation. This article serves as a valuable guide to help researchers select high-quality antibodies for their specific needs, facilitating the biochemical and cellular assessment of sFRP-1 properties and function.

## Results and discussion

Our standard protocol involves comparing readouts from wild-type and knockout (KO) cells. ^9^^,^^10^ The first step is to identify a cell line(s) that expresses sufficient levels of a given protein to generate a measurable signal. To this end, we examined the DepMap transcriptomics database to identify all cell lines that express the target at levels greater than 2.5 log _2_ (transcripts per million “TPM” +1), which we have found to be a suitable cut-off (Cancer Dependency Map Portal, RRID:SCR_017655). Commercially available A549 cells expressed the sFRP-1 transcript at RNA levels above the average range of cancer cells analyzed. Parental and *SFRP1* knockout A549 cells were obtained from Abcam ( Table 1).

**Table 1.** Summary of the cell lines used.

Institution	Catalog number	RRID (Cellosaurus)	Cell line	Genotype
Abcam	ab275463	CVCL_0023	A549	WT
Abcam	ab277906	CVCL_B2Q1	A549	*SFRP1* KO

**Figure 1.**
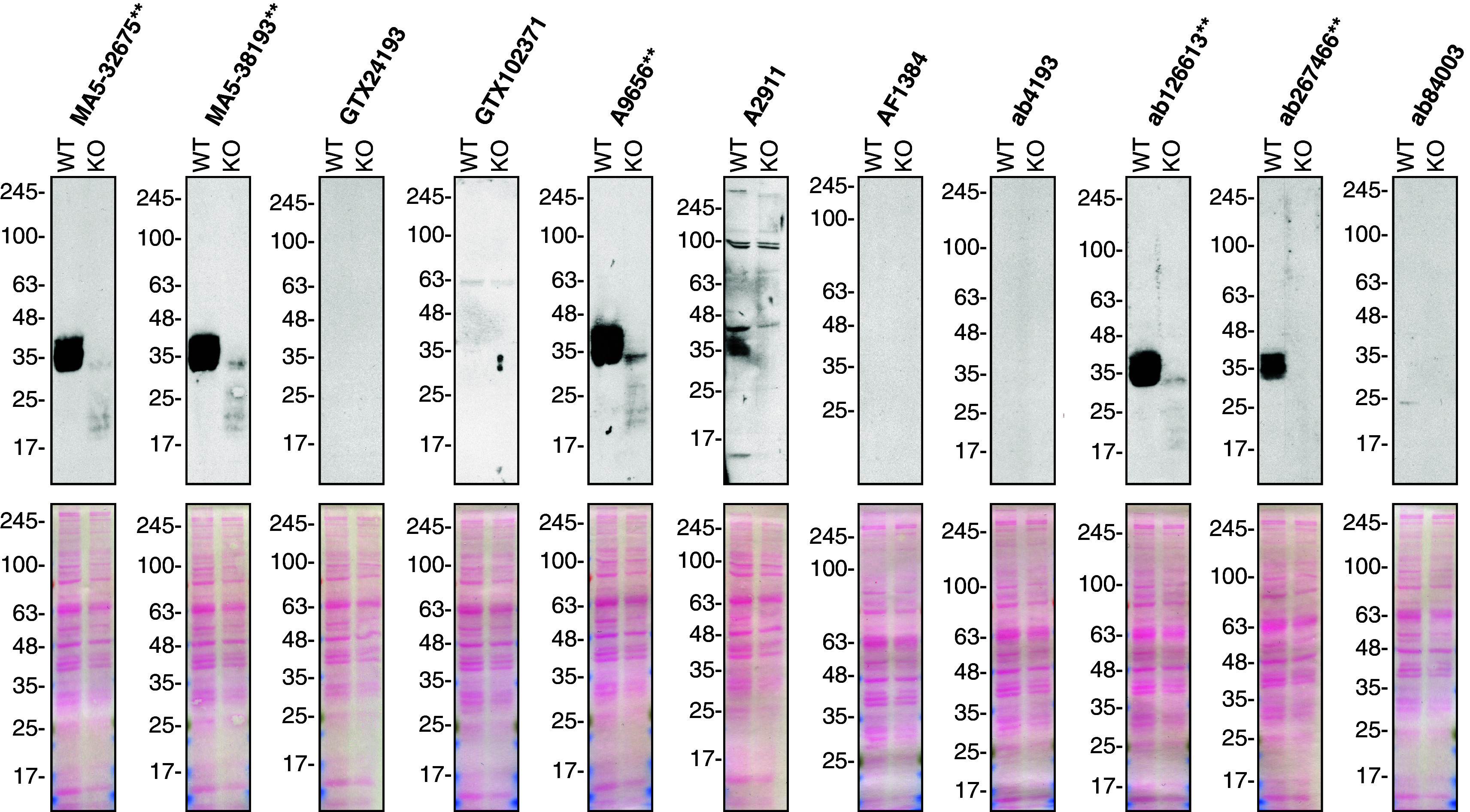
Secreted frizzled-related protein 1 antibody screening by Western Blot on culture media. A549 (WT and *SFRP1* KO) were treated with Brefeldin A at 3.0 μg/ml for 18 hrs. 50 μg of protein from concentrated culture media were processed for Western Blot with the indicated sFRP-1 antibodies. The Ponceau stained transfers of each blot are shown. Antibody dilutions were chosen according to the recommendations of the antibody supplier. Antibody dilution used: MA5-32675** at 1/500; MA5-38193** at 1/500; GTX24193 at 1/1000; GTX102371 at 1/1000; A9656** at 1/1000; A2911 at 1/1000; AF1384 at 1/500; ab4193 at 1/500; ab126613** at 1/1000; ab267466** at 1/1000; ab84003 at 1/1000. Predicted band size: 35 kDa. **Recombinant antibody.

**Figure 2.**
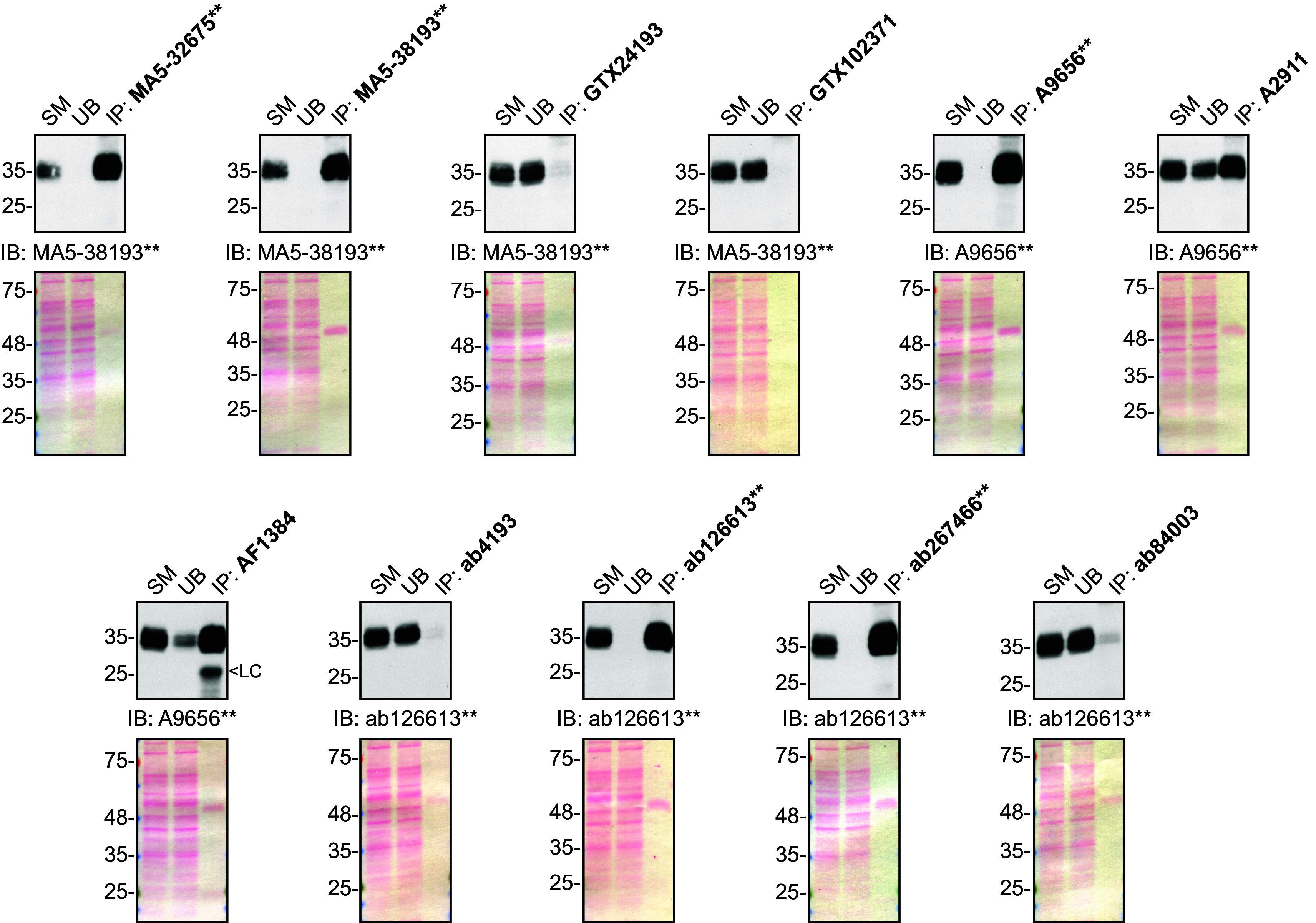
Secreted frizzled-related protein 1 antibody screening by immunoprecipitation on culture media. Immunoprecipitation was performed on concentrate culture media using 1.0 μg of the indicated sFRP-1 antibodies pre-coupled to either protein A or protein G magnetic beads. Samples were washed and processed for Western Blot with the indicated sFRP-1 antibody. For Western Blot, MA5-38193** was used at 1/500, A9656** at 1/1000 and ab126613** at 1/1000. The Ponceau stained transfers of each blot are shown for similar reasons as in Figure 1. SM = 10% starting material; UB = 10% unbound fraction; IP = immunoprecipitated; LC = light chain. **Recombinant antibody.

SFRP-1 is predicted to be a secreted protein. Accordingly, we collected concentrated culture media from both wild-type and *SFRP1* KO cells and used the conditioned media to probe the performance of the antibodies ( Table 2) side-by-side by Western blot and immunoprecipitation. The profiles of each of the antibodies are shown in Figures 1 and 2. The datasets can be found as *Underlying data.* ^13^^,^^14^

**Table 2.** Summary of the Secreted frizzled-related protein 1 antibodies tested.

Company	Catalog number	Lot number	RRID (Antibody Registry)	Clonality	Clone ID	Host	Concentration (μg/μl)	Vendors recommended applications
Thermo Fisher Scientific	MA5-32675 **	WJ3417745	AB_2809952	recombinant-mono	JA11-68	rabbit	1.00	Wb
Thermo Fisher Scientific	MA5-38193 **	WJ3417799B	AB_2898110	recombinant-mono	ARC1683	rabbit	0.88	Wb
GeneTex	GTX24193	822102161	AB_370619	polyclonal	-	rabbit	1.00	Wb, IF
GeneTex	GTX102371	39911	AB_1951886	polyclonal	-	rabbit	0.33	Wb
ABclonal	A9656 **	4000001683	AB_2863750	recombinant-mono	ARC1683	rabbit	0.88	Wb
ABclonal	A2911	31570101	AB_2764730	polyclonal	-	rabbit	2.68	Wb
Bio-Techne	AF1384	IRQ1020021	AB_2285831	polyclonal	-	goat	0.20	Wb, IF
Abcam	ab4193	GR3345186-4	AB_304357	polyclonal	-	rabbit	1.00	Wb, IF
Abcam	ab126613 **	GR3350102-3	AB_11128257	recombinant-mono	EPR7003	rabbit	0.41	Wb
Abcam	ab267466 **	GR3321068-3	AB_2904616	recombinant-mono	EPR23092-253	rabbit	0.46	Wb, IP
Abcam	ab84003	GR42188-1	AB_10670402	polyclonal	-	rabbit	1.00	Wb

Wb = Western blot; IF = immunofluorescence; IP = immunoprecipitation.

** Recombinant antibody.

In conclusion, we have screened eleven sFRP-1 commercial antibodies by Western Blot and immunoprecipitation. Several high-performing antibodies that successfully detect sFRP-1 under our standardized experimental conditions. In our effort to address the antibody reliability and reproducibility challenges in scientific research, the authors recommend the antibodies that demonstrated to be underperforming under our standard procedure be removed from the commercial antibody market. However, the authors do not engage in result analysis or offer explicit antibody recommendations. A limitation of this study is the use of universal protocols – any conclusions remain relevant within the confines of the experimental setup and cell line used in this study. Our primary aim is to deliver top-tier data to the scientific community, grounded in Open Science principles. This empowers experts to interpret the characterization data independently, enabling them to make informed choices regarding the most suitable antibodies for their specific experimental needs.

## Methods

### Antibodies

All sFRP-1 antibodies are listed in Table 2. Peroxidase-conjugated goat anti-rabbit and donkey anti-goat antibodies are from Thermo Fisher Scientific (cat. number 65-6120 and A15999).

### Cell culture

A549 WT and *SFRP1* KO used are listed in Table 1. Cells were cultured in DMEM high-glucose (GE Healthcare cat. number SH30081.01) containing 10% fetal bovine serum (Wisent, cat. number 080450), 2 mM L-glutamate (Wisent cat. number 609065), 100 IU penicillin and 100 μg/ml streptomycin (Wisent cat. number 450201). Cells were starved in DMEM high-glucose containing L-glutamate and penicillin/streptomycin.

### Antibody screening by Western Blot on culture media

A549 cells (WT and *SFRP1* KO) were washed 3× with PBS and starved for ~18 hrs. Culture media were collected and centrifuged for 10 min at 500 × g to eliminate cells and larger contaminants, then for 10 min at 4500 × g to eliminate smaller contaminants. Culture media were concentrated by centrifuging at 4000 × g for 10 min using Amicon Ultra-15 Centrifugal Filter Units with a membrane NMWL of 10 kDa (MilliporeSigma cat. number UFC901024).

Western Blots were performed as described in our standard operating procedure. ^11^ Western Blots were performed with large 8-16% gradient polyacrylamide gels and transferred on nitrocellulose membranes. Proteins on the blots were visualized with Ponceau staining which is scanned to show together with individual Western blot. Blots were blocked with 5% milk for 1 hr, and antibodies were incubated overnight at 4°C with 5% bovine serum albumin in TBS with 0,1% Tween 20 (TBST). Following three washes with TBST, the peroxidase conjugated secondary antibody was incubated at a dilution of ~0.2 μg/ml in TBST with 5% milk for 1 hr at room temperature followed by three more washes with TBST. Membranes were incubated with ECL from Pierce (cat. number 32106) prior to detection with HyBlot CL autoradiography films from Denville (cat. number 1159T41).

### Antibody screening by immunoprecipitation on culture media

Immunoprecipitation was performed as described in our standard operating procedure. ^12^ Antibody-bead conjugates were prepared by adding 1 μg of antibody to 500 ul of Pierce IP Buffer from Thermo Fisher Scientific (cat. number 87788) in a microcentrifuge tube, together with 30 μl of Dynabeads protein A - (for rabbit antibodies) or protein G - (for goat antibodies) from Thermo Fisher Scientific (cat. number 10002D and 10004D, respectively). Pierce IP Lysis Buffer (25 mM Tris-HCl pH 7.4, 150 mM NaCl, 1 mM EDTA, 1% NP-40 and 5% glycerol) was supplemented with the Halt Protease Inhibitor Cocktail 100X from Thermo Fisher Scientific (cat. number 78446) at a final concentration of 1×. Tubes were rocked for ~2 hrs at 4°C followed by two washes to remove unbound antibodies.

Starved A549 WT culture media were concentrated as described above. 1 ml aliquots at 0.5 mg/ml of lysate were incubated with an antibody-bead conjugate for ~2 hrs at 4°C. Following centrifugation, the unbound fractions were collected, and beads were subsequently washed three times with 1.0 ml of IP Lysis Buffer and processed for SDS-PAGE and Western Blot on 8-16% polyacrylamide gels. Prot-A: HRP (MilliporeSigma, cat. number P8651) was used as a secondary detection system at a dilution of 0.4 μg/ml.

## Data Availability

Zenodo: Antibody Characterization Report for Secreted frizzled-related protein 1, https://doi.org/10.5281/zenodo.6370454. ^13^ Zenodo: Dataset for the Secreted frizzled-related protein 1 antibody screening study, https://doi.org/10.5281/zenodo.7571170. ^14^ Data are available under the terms of the Creative Commons Attribution 4.0 International license (CC-BY 4.0).
