# Supplementary material for: A guide to selecting high-performing antibodies for Secreted frizzled-related protein 1 (sFRP-1) for use in Western Blot and immunoprecipitation
Source: F1000Res. 2024 Apr 2;12:291. Originally published 2023 Mar 16. [Version 2] doi: 10.12688/f1000research.130991.2 (PMC11420615; doi:10.12688/f1000research.130991.2)
Supplement: Peer review history

# Reviewer report: Reviewer response for version 2

**DOI:** 10.5256/f1000research.164273.r325242

**Published:** 2024-09-23

**Authors:** Lynne Howells

**Affiliations:** 1 University of Leicester, Leicester, UK

The article describes a protocol that will facilitate the use of better antibody tools in the detection of the sFRP-1 protein via western blot and IP techniques.

 The article is succinct, well written and will provide an enabler for researchers looking to analyse this specific protein.

 Even though the article is short, there are a number of areas where 'lab slang' has crept in, which should be rectified.:

 When saying cells were starved – specify serum starved, or say that media was subsequently replaced with serum-free media.

 In the Ab screening by IP section – it says 500 uL instead of micro (mu symbol)

 Millilitres should  be mL rather than ml

Are sufficient details of methods and materials provided to allow replication by others?

Yes

Is the rationale for creating the dataset(s) clearly described?

Yes

Are the datasets clearly presented in a useable and accessible format?

Yes

Are the protocols appropriate and is the work technically sound?

Yes

Reviewer Expertise:

Cancer biology, cell signalling (WB, IP, FACS, IHC, IF)

I confirm that I have read this submission and believe that I have an appropriate level of expertise to confirm that it is of an acceptable scientific standard.

# Reviewer report: Reviewer response for version 2

**DOI:** 10.5256/f1000research.164273.r261793

**Published:** 2024-04-18

**Authors:** Oi Wah Liew

**Affiliations:** 1 Cardiovascular Research Institute, Department of Medicine, Yong Loo Lin School of Medicine, National University of Singapore, National University Health System, Singapore, Singapore

The authors have not addressed any of my concerns. However, I note that they have made a qualifying statement in their report that "any conclusions drawn remain relevant within the confines of the experimental set up and cell line used in this study." I accept that those in the field will be able to interpret the available data in the light of their expertise.

 As such, I understand that the authors need only to provide information on why and how the data were created but do not need to include analyses or conclusions. Indeed, the authors did not address any of my concerns, some of which 

 requires additional experiments or an explanation of seemingly contradictory data between Figure 1 and 2. Since the instructions to authors is that analyses or conclusions are not required, I am not sure if I am asking more than is required of the authors. It would be great if the authors could at least provide a point-by-point rebuttal of my concerns in the comments section.

Are sufficient details of methods and materials provided to allow replication by others?

Yes

Is the rationale for creating the dataset(s) clearly described?

Yes

Are the datasets clearly presented in a useable and accessible format?

Yes

Are the protocols appropriate and is the work technically sound?

Partly

Reviewer Expertise:

Biomarkers, antibody generation/characterization and validation, antigen design, recombinant protein expression, genetic engineering, assay development.

I confirm that I have read this submission and believe that I have an appropriate level of expertise to confirm that it is of an acceptable scientific standard, however I have significant reservations, as outlined above.

# Response

**Published:** 2024-05-23

**Authors:** Kathleen Southern

**Affiliations:** McGill University, Canada

Please see below our response to your concerns, point-by-point. We hope these responses address any misunderstandings or hesitations you previously had, providing a clearer  understanding of the manuscripts purpose, the YCharOS initiative as a whole and limitations we face in terms of data interpretation and our protocol.

 1.       The authors selected the A549 cell line based on available data on high expression at transcript levels. However, high transcript levels do not necessarily translate to high protein expression. The A549 and A549 sFRP1 KO cell lines as well as some of the antibodies tested were from the same vendor. To have added confidence that the selected antibodies are recognizing endogenous sFRP-1 expressed in A549 cells, the authors should include a recombinant source of sFRP-1 from a different vendor in their western blots. In others words, each antibody is tested against endogenous sFRP-1 expressed in A549 cells, A549 SFRP1 KO (negative control) and recombinant sFRP-1 (eg R&D Systems cat no. 1384-SF).

 Agreed, mRNA levels do not always reflect protein expression, however our results show that A549 is an appropriate cell line for antibody testing as the protein was very easily detected in the culture medium by western blot.

 The antibodies tested are from five different vendors as shown in Table 2: Thermo Fisher Scientific, GeneTex, ABclonal, Bio-Techne and Abcam.

 The purpose of this manuscript is to detect the endogenous protein in a relevant sample (culture medium because the protein is secreted). Testing the antibody’s ability to recognize the recombinant proteins is screened internally and routinely by the manufacturer but do not always mean recognition in biologically relevant samples that researchers may be interested in investigating. This study offers a valuable antibody screening in the appropriate sample and in the presence of the most agreed upon type of negative control, a KO.

 2.       Human sFRP-1 is a member of the SFRP family. Since many of the antibodies selected are polyclonal in nature, the authors should assess potential cross-reactivity against other closely related family members eg human sFRP-2, sFRP-4 and sFRP-5. Sequence alignment between sFRP-1 against these family members shows stretches of 100% identical amino acids up to 22 amino acids long, portending potential cross-reactivity of anti-sFRP-1 polyclonal antibodies against these entities. Cross-reactivity of the recombinant monoclonal antibodies should also be tested since there is no epitope information for these antibodies.

 Cross-reactivity against other closely related family members can only be assessed using other KO controls corresponding to the mentioned genes of interest which we do not have access to. For now, this work is only informative about antibody specificity against sFRP1.

 3.       The results for GTX4193, GTX102371, AF1384, AB4193 and AB84003 in Figure 1 and 2 appear contradictory. In Figure 1, these antibodies could not detect sFRP-1 present in concentrated culture media collected from starved A549 cells by western blotting. However, these antibodies were able to detect sFRP-1 at ~35 kDa position in the starting material samples in Figure 2 which are essentially equivalent to the WT samples in Figure 1.

 In Figure 2, as indicated in the legend, all starting material and immunoprecipitates corresponding to each antibody tested are western blotted with one antibody, MA5-38193. Since MA5-38193 detects sFRP-1 by WB (Figure 1), signal is visible in all SM lanes.

 The detailed antibody characterization platform we have created, along with academic and industry researchers, is available on Protocol Exchange. https://doi.org/10.21203/rs.3.pex-2607/v1

# Reviewer report: Reviewer response for version 1

**DOI:** 10.5256/f1000research.143794.r244759

**Published:** 2024-02-22

**Authors:** Oi Wah Liew

**Affiliations:** 1 Cardiovascular Research Institute, Department of Medicine, Yong Loo Lin School of Medicine, National University of Singapore, National University Health System, Singapore, Singapore

Comments to the manuscript “The identification of high-performing antibodies for Secreted  frizzled-related protein 1 (sFRP-1) for use in Western blot and immunoprecipitation”;

 Ayoubi R, Southern K, Laflamme C and NeuroSGC/YCharOS collaborative group

 GENERAL COMMENT

 The manuscript describes the performance of 10 different commercial antibodies (rabbit or goat polyclonal and recombinant monoclonal antibodies) for detecting secreted frizzled-related protein 1 (sFRP-1) for Western blot and immunoprecipitation applications. A high expressing commercial cell line, A549, and its sFRP-1 KO counterpart was used as positive and negative controls for testing.   

 The work and its findings have scientific merits; however, there are several specific points that should be addressed by the authors. These are provided below:

**SPECIFIC COMMENTS**

 1.       The authors selected the A549 cell line based on available data on high expression at transcript levels. However, high transcript levels do not necessarily translate to high protein expression. The A549 and A549 sFRP1 KO cell lines as well as some of the antibodies tested were from the same vendor. To have added confidence that the selected antibodies are recognizing endogenous sFRP-1 expressed in A549 cells, the authors should include a recombinant source of sFRP-1 from a different vendor in their western blots. In others words, each antibody is tested against endogenous sFRP-1 expressed in A549 cells, A549 SFRP1 KO (negative control) and recombinant sFRP-1 (eg R&D Systems cat no. 1384-SF).

 2.       Human sFRP-1 is a member of the SFRP family. Since many of the antibodies selected are polyclonal in nature, the authors should assess potential cross-reactivity against other closely related family members eg human sFRP-2, sFRP-4 and sFRP-5. Sequence alignment between sFRP-1 against these family members shows stretches of 100% identical amino acids up to 22 amino acids long, portending potential cross-reactivity of anti-sFRP-1 polyclonal antibodies against these entities. Cross-reactivity of the recombinant monoclonal antibodies should also be tested since there is no epitope information for these antibodies.

 3.       The results for GTX4193, GTX102371, AF1384, AB4193 and AB84003 in Figure 1 and 2 appear contradictory. In Figure 1, these antibodies could not detect sFRP-1 present in concentrated culture media collected from starved A549 cells by western blotting. However, these antibodies were able to detect sFRP-1 at ~35 kDa position in the starting material samples in Figure 2 which are essentially equivalent to the WT samples in Figure 1.

Are sufficient details of methods and materials provided to allow replication by others?

Yes

Is the rationale for creating the dataset(s) clearly described?

Yes

Are the datasets clearly presented in a useable and accessible format?

Yes

Are the protocols appropriate and is the work technically sound?

Partly

Reviewer Expertise:

Biomarkers, antibody generation/characterization and validation, antigen design, recombinant protein expression, genetic engineering, assay development.

I confirm that I have read this submission and believe that I have an appropriate level of expertise to confirm that it is of an acceptable scientific standard, however I have significant reservations, as outlined above.

# Response

**Published:** 2024-03-25

**Authors:** Kathleen Southern

**Affiliations:** McGill University, Canada

Thank you Oi Wah Liew for your peer review report!

 To address your first comment, we are aware that RNA transcript and protein levels do not always correlate. That being said, we have found that an RNA expression value of 2.5 log _2_(TPM+1) serves as an appropriate minimal threshold level that will yield detectable protein levels in antibody screening. We understand that this method has its limitations, and in the case that we were unable to detect a strong signal of sFRP-1 in the WT, we have an alternative protocol. A further validation strategy is used to help select the optimal cell line by selecting 4-8 cancer cell lines with high RNA scores to assess and compare protein expression in cell lines in WB. Although outside the scope of our standardized protocol, we would recommend to researchers to use this study as a guide to select high-quality antibodies, then validate the antibodies using a commercial recombinant sFRP-1, as suggested.

 The evaluation of cross reactivity of the sFRP-1 antibodies is, once again, outside the scope of this study. We leave these subsequent studies up to the experts studying the sFRP family of proteins. This study is part of a broader collaborative initiative aimed at validating commercial antibodies for every human protein, preventing us from performing follow up studies for every protein target.

 To address your final comment, in Western blot, 50 μg of sample was processed onto the gel l whereas in Immunoprecipitation (IP), 100 μg of protein sample was processed. This data is found the Figure legends. This can explain the antibodies ability to detect a more concentrated amount of proteins and generate a signal in the starting material in Figure 2 as compared to Figure 1. Regardless, a successful antibody should enrich its target in the IP compared to the starting material, and deplete it from the unbound fraction, as seen for antibody MA5-32675**.

 For more information on how to interpret the antibody characterization data in this study, an editorial by Biddle et al., featured on our YCharOS gateway, can serve as an excellent guide. https://f1000research.com/articles/12-1344

 To address comments from another reviewer’s report, a second version of this Data Note has been submitted to F1000. We encourage you to review this subsequent version as well as we believe it will help address some of your comments and provide a clearer definition of the YCharOS initiative.

# Reviewer report: Reviewer response for version 1

**DOI:** 10.5256/f1000research.143794.r185117

**Published:** 2024-02-08

**Authors:** Viyatprajna Acharya

**Affiliations:** 1 KIMS, KIIT, Bhubaneswar, India

The result can be discussed a little more elaborately. Methods are well-explained. Supporting diagram for immunoprecipitation and Western blot may be provided. The conclusion can be more explicit such as describing how many samples were actually suitable or better over others. References 8-11 are incomplete.

 The article is well-written and maybe considered for indexing after minor corrections.

Are sufficient details of methods and materials provided to allow replication by others?

Yes

Is the rationale for creating the dataset(s) clearly described?

Yes

Are the datasets clearly presented in a useable and accessible format?

Partly

Are the protocols appropriate and is the work technically sound?

Yes

Reviewer Expertise:

Immunochemistry, metabolomics, cancer, oxidative stress.

I confirm that I have read this submission and believe that I have an appropriate level of expertise to confirm that it is of an acceptable scientific standard, however I have significant reservations, as outlined above.

# Response

**Published:** 2024-03-25

**Authors:** Kathleen Southern

**Affiliations:** McGill University, Canada

Dear Viyatprajna Acharya,

 Thank you for submitting a peer review report for this Data Note.

 An open-access Dataset is available under the “Data Availability” section. You can find the supporting diagrams and underlying data for Western blot and immunoprecipitation experiments within this dataset. As an organization that prioritizes open science, we ensure that all data is transparent and readily available which is why all of our publications have equivalent Datasets.

 The objective of this article is not to interpret the results nor score the performance of the antibodies. Given that it is formatted as a Data Note, it does not require the results to be discussed or concluded. YCharOS is a public-private organization whose mission is to characterize antibodies for every human protein and deliver the research as a collective good for the scientific community. That being said, we understand how this intention may be misinterpreted. A new version has been submitted, to include modifications to the title, introduction and results&discussion section that ensure our goals clearly are aligned and defined to all viewers.

 References 8-11 have been edited and will be complete in the newly submitted version.

 Thank you for your feedback and corrections!
